# Successful replacement of the longest worldwide *in situ* Nanostim leadless cardiac pacemaker for a Micra Transcatheter Pacing System

**DOI:** 10.1007/s10840-017-0310-2

**Published:** 2018-01-15

**Authors:** Niek E. G. Beurskens, Fleur V. Y. Tjong, Anne-Floor B. E. Quast, Reinoud E. Knops

**Affiliations:** AMC Heart Center, Department of Cardiology, Academic Medical Center, University of Amsterdam, Meibergdreef 9, 1105 AZ Amsterdam, The Netherlands

**Keywords:** Leadless cardiac pacing, End-of-life management, Retrieval, Nanostim leadless cardiac pacemaker, Micra Transcatheter Pacing System

The potential inability to retrieve chronically implanted leadless pacemakers (LP) at end-of-life limits the application of this novel technology. Data on safe long-term LP retrieval is lacking, yet is of paramount importance. We present the case of an 80-year-old patient in whom a Nanostim LP was successfully implanted for chronic atrial fibrillation with symptomatic slow ventricular response in January 2013. Background history was notable for rheumatic aortic and mitral stenosis and status post mechanical aortic and mitral valve replacement. At a regular follow-up visit 4 years and 9 months after LP implantation, the initial normal communication with the device was lost during impedance measurement, a known trigger for battery failure. The patient had 73% right ventricular pacing. Strategies to replace LPs reaching end of service remain an unsettled concern. Suggested strategies comprise the following: placing an additional leadless device adjacent to the LP or retrieving the non-functioning LP and subsequently implanting a new device. We decided to extract the LP in order to limit the amount of non-functioning intracardiac hardware and mitigate the potential device-device interference and unknown long-term risks of multiple intracardiac devices. In addition, retrieval may result in a more accessible right ventricle (RV) in case re-implantation of a new device is indicated. The procedure was performed in the catheterization laboratory under fluoroscopic guidance and local anesthesia (Fig. 1). The retrieval catheter (Abbott) was percutaneously introduced via the femoral vein by using a 27 French introducer sheath (Medtronic). The single-loop snare and the integrated protective sleeve at the end of the retrieval catheter were engaged towards the RV. The snare was engaged to capture the distal cap of the device. After docking the device, the helix was unscrewed from the endocardium. The protective sleeve was advanced over the LP and subsequently the device was removed. A Micra Transcatheter Pacing System (TPS) was inserted by the catheter delivery system (Medtronic) through the right femoral vein with the use of the same 27 French introducer (Medtronic). The Micra TPS was fixated to the myocardium in the RV apex at a more septal position. The Micra TPS implantation was completed without complications and with stable electrical parameters (i.e., RV sensing of > 20 mV, impedance of 740 Ω and a pacing threshold of 0.38 Volts at 0.24 ms). Histopathological examination showed minimal adherent fibrous tissue at the proximal docking feature and helix of the Nanostim. We showed a safe and easy extraction of the longest worldwide *in situ* Nanostim. The feasibility of chronic uneventful LP retrievals, if confirmed by subsequent trials or follow-up studies, will demonstrate that there is an effective end-of-life device strategy, which makes LP therapy a viable alternative to standard lead-based pacing. Considering the increasing incidence of patients in whom the Nanostim battery fails to meet their projected longevity, this information is highly relevant for all physicians implanting these devices.

**Fig. 1:**
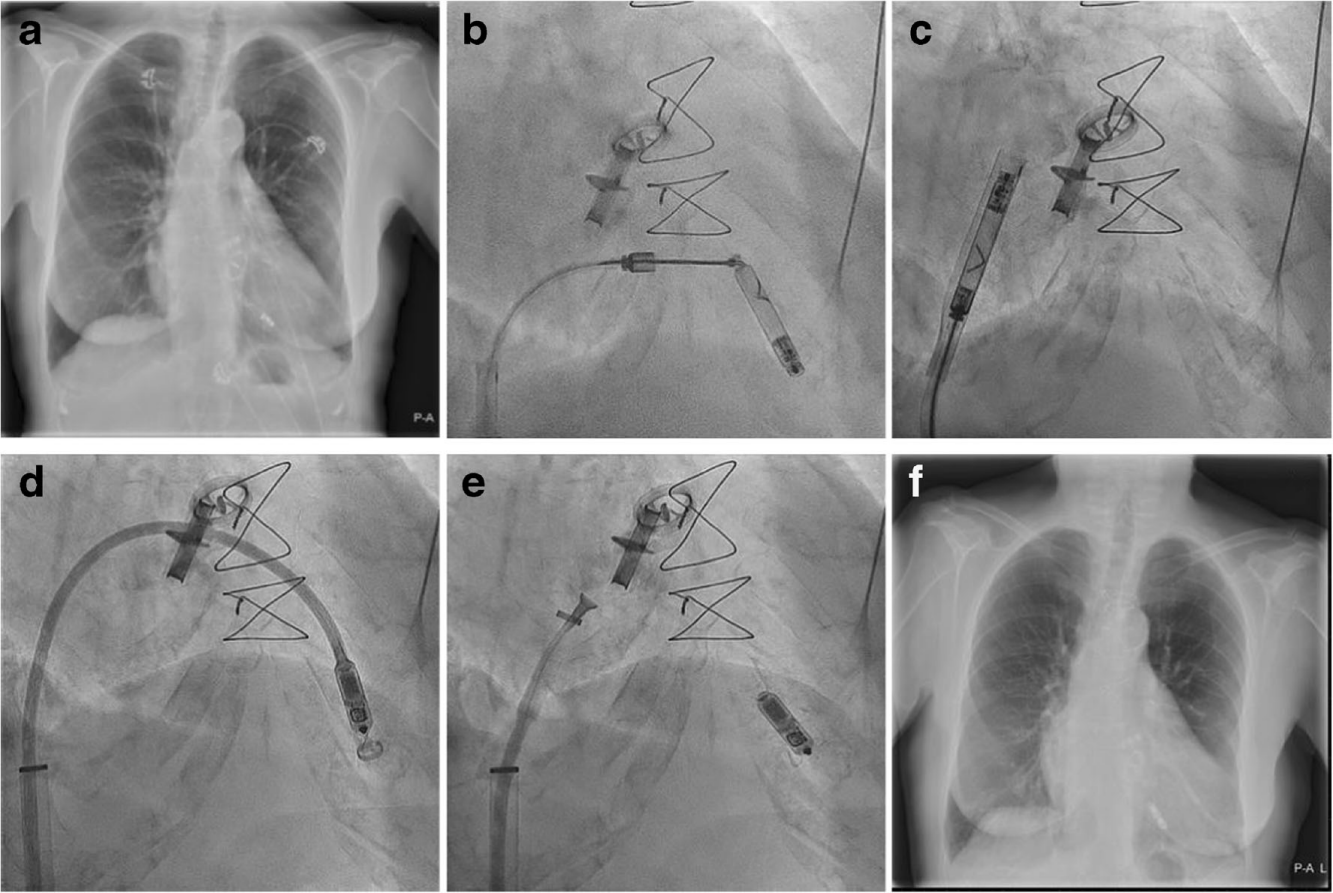
**a** Chest radiography of the *in situ* Nanostim. **b** Capture of the distal cap of the device by the snare at the end of the retrieval catheter. **c** Nanostim removal from the RV. **d** Micra TPS insertion in the RV. **e** Micra fixation in the right ventricular myocardium at a slightly different location (i.e., more septal) with the nitinol tines. **f** Chest radiography of the *in situ* Micra TPS

## Electronic supplementary material

ESM 1 (MP4 10,103 kb)

